# Supplementary material for: Which programmes and policies across health and community settings will generate the most significant impacts for youth suicide prevention in Australia and the UK? Protocol for a systems modelling and simulation study
Source: BMJ Open. 2023 Aug 14;13(8):e071111. doi: 10.1136/bmjopen-2022-071111 (PMC10432673; doi:10.1136/bmjopen-2022-071111)
Supplement: Supplementary data [file bmjopen-2022-071111supp001.pdf]

REF: 22885

Young People's Interview Topic Guide

Project: SEYMOUR: System Dynamics Modelling for Suicide Prevention

Introduction

My name is XXX.
Thank you for agreeing to participate in this study.

Purpose:

"As I explained before, we are a team of researchers at Orygen, University of Melbourne and we have developed a model that can help us decide which interventions can help reduce rates of suicide & attempted suicide among young people aged 12-25 in North Western Melbourne.

To do this, we worked in partnership over the past few months with young people with lived experience of self-harm and/or suicidal behaviour, carers, health professionals and youth advocacy organisations supporting young people with lived experience of self-harm and/or suicidal behaviour. We also used data already collected through a wide variety of sources (e.g. Australia Bureau of Statistics) to help us test if and how well the model works; and, which interventions or programmes work best in reducing rates of suicide and attempted suicide among young people aged 12-25 in North Western Melbourne.

These tests showed that the most promising interventions are [enter].

What we would like to do now is ask young people for their views and perspectives of how best to put these interventions in practice; what could help or hinder the rollout of these interventions.

Themes/topics to be explored

1. Perceived value and interest in [enter intervention]
2. Specific role that young people can play in facilitating the adoption/implementation of [enter intervention]
3. Barriers to successful implementation of [enter intervention]
   Prompts: Ask about cultural/socioeconomic barriers, barriers in relation to the setting, context, organisation etc.

Michail M, *et al. BMJ Open* 2023; 13:e071111. doi: 10.1136/bmjopen-2022-071111

REF: 22885

4. Facilitators to successful implementation of [enter intervention]
   Prompts: Ask about facilitators in relation to the setting, context, population (i.e. young people), organisation etc.
5. Perceived successes and challenges experienced [relevant only if a young person has in the past received the intervention]
6. Recommendations for future roll-out of [enter intervention] in North Western Melbourne

Wrap-up questions

1. Is there anything else you would like to say that we have not covered?
2. Do you have any questions for me?

Thank you for taking part in this study.

Interview Topic Guide for Policy Makers, Service Planners and Ministerial Advisors

Project: *SEYMOUR: System Dynamics Modelling for Suicide Prevention*

Introduction

My name is [insert name]. Thank you for agreeing to participate.

Purpose:

We are a team of researchers at Orygen, University of Melbourne and we have developed a model that can help us decide which interventions can help reduce rates of suicide & attempted suicide among young people aged 12-25 in North Western Melbourne.

To do this, we worked in partnership over the past few months with young people with lived experience of self-harm and/or suicidal behaviour, carers, health professionals (e.g. GPs, psychiatrists, nurses), youth advocacy organisations supporting young people with lived experience of self-harm and/or suicidal behaviour; and local healthcare policy makers. We also used data already collected through a wide variety of sources (e.g. Australia Bureau of Statistics) to help us test if and how well the model works. This has

REF: 22885

helped us identify which interventions or programmes work best in reducing rates of suicide and attempted suicide among young people aged 12-25 in North Western Melbourne.

Based on our findings, the most promising interventions are [enter].

This project aims to the views of local/regional *suicide prevention policy leads, service commissioners; health and social care ministerial advisors and policy makers across Victoria [delete as appropriate] about the implementation and sustainable embedding of those suicide prevention interventions in North Western Melbourne. We are interested in your views on contextual, procedural and other factors that can influence the adoption and implementation of those interventions in practice.*

Do you have any questions before we start?

Themes/topics to be explored

7. Perceived value, relevance and interest in [enter intervention]
8. Specific role that [enter role of professional interviewed] can play in facilitating the adoption/implementation of [enter intervention]
9. Barriers to successful implementation of [enter intervention]
   Prompts: Ask about barriers in relation to individual, setting, context, organisation, resources, socio-political factors [where relevant], the intervention itself etc.
10. Facilitators to successful implementation of [enter intervention]
    Prompts: Ask about facilitators in relation to the individual, setting, context, organisation, resources, socio-political factors [where relevant], the intervention itself etc.
11. Perceived successes and challenges experienced [relevant only if professional interviewed has in the past been involved in the adoption/implementation of the intervention]
12. Recommendations for future roll-out of [enter intervention] in North Western Melbourne

Wrap-up questions

3. Is there anything else you would like to say that we have not covered?
4. Do you have any questions for me?

Thank you for taking part in this study.

REF: 22885
