# Supplementary material for: Which programmes and policies across health and community settings will generate the most significant impacts for youth suicide prevention in Australia and the UK? Protocol for a systems modelling and simulation study
Source: BMJ Open. 2023 Aug 14;13(8):e071111. doi: 10.1136/bmjopen-2022-071111 (PMC10432673; doi:10.1136/bmjopen-2022-071111)
Supplement: Supplementary data [file bmjopen-2022-071111supp002.pdf]

QUALTRICS SURVEY

SECTIONS

1. **Basic demographic information:**
   - age
   - gender
   - ethnicity
   - occupation
2. **Barriers to implementation**
   A list of barriers (identified via interviews/focus groups) will be presented along with a brief description of each barrier.
   "*Please accept or reject each barrier depending on how relevant or important you think it is*"

3. **Implementation strategies/plans**
   - For each accepted barrier, participants will be presented with a range of strategies (based on the CFIR-ERIC Matching Tool) and will be asked to select and rank those strategies/plans, from the list below, you believe to be more important in addressing the specific barrier (e.g., #1 is the top strategy).
   - A free-text box will be provided so that participants can add an explanation or any other information they wish.
4. **Feasibility, improvement opportunity, validity, difficulty, relevance**
   The final section of the survey will ask participants to report using a 3-point Likert scale (0=not influential; 1= somewhat influential; 2= extremely influential) to what extent feasibility (i.e. *can the strategy realistically be applied to the barrier*?); improvement opportunity (i.e. *will this strategy make a big impact*?); validity (i.e. *is the evidence base for the strategy compelling*?); difficulty (i.e. *what are the work and resource requirements for the strategy*?); and relevance (i.e. *does the strategy have direct relevance to the barrier*?) of each implementation strategy influenced their ranked choices.
