# Supplementary material for: Which programmes and policies across health and community settings will generate the most significant impacts for youth suicide prevention in Australia and the UK? Protocol for a systems modelling and simulation study
Source: BMJ Open. 2023 Aug 14;13(8):e071111. doi: 10.1136/bmjopen-2022-071111 (PMC10432673; doi:10.1136/bmjopen-2022-071111)
Supplement: Supplementary data [file bmjopen-2022-071111supp003.pdf]

SEYMOUR STUDY

PARTICIPATORY MODELLING WORKSHOPS

RISK MANAGEMENT STRATEGY

- Briefing session where young people will complete a wellness plan including the details of their support person/clinician; triggers and strategies they use when feeling upset.
- Debriefing session where young people can reflect on the workshops and a debrief sheet containing information about the study, signposting information and contact information for suicide prevention helplines.
- Clinical support on stand-by throughout the workshops. Any potential escalation of risk will be dealt with on a case-by-case basis. Confidentiality and its limits will be explained in the Participant Information Sheet.
- If a participant becomes distressed during a workshop, they will be given the opportunity to have a break or withdraw from the study completely. If the participant appears to be extremely distressed or communicates current suicide risk, the interviewer will conduct a brief risk assessment. If the participant is not at risk, but remains distressed, the interviewer will encourage them to access support, offer to contact the participant's support person or parent/guardian, and/or help them engage in their pre-specified stress management technique/s (if relevant). If the participant is at immediate risk, the interviewer will contact emergency services (and/or, for participants under 18, their parent/guardian). A member of the research team will check-in either via email or text message within 48 hours.
- Disclosure of safeguarding concerns: If during the course of the research, the research team becomes aware of a child or vulnerable adult being at harm or at risk of harm, they will immediately notify their GP (and parent/legal guardian in case of minors) in order to follow relevant protocols to log concerns with their organisations.
- Disclosure of suicide risk (intent, thoughts, behaviour): All information obtained during the course of the research will be kept strictly confidential unless the research team has reasons to be concerned about the safety or wellbeing of a young person. If this is the case, the team will contact their GP or primary clinician; and inform parents/legal guardians in case of minors.
- Participants may experience negative power dynamics: The workshops will involve a range of experts including experts by experience (young people including minors); research experts (research team); experts in delivering services and/or supporting to young people with lived experience of suicidality. The research team will clarify the ground rules of the workshop at the beginning of each session highlighting the different and complementary types of expertise in the workshop; the importance of listening respectfully and carefully to one another; ensuring that everyone has the chance to speak if they want to and be mindful of dominating discussions. In order to manage power dynamics and facilitate the meaningful involvement of all participants and particularly those with lived experience, we will ensure that:
  - Young people will be briefed prior to the workshops and we will provide young people with the agenda beforehand to give them the opportunity to prepare.
  - Young people will have the opportunity to share their thoughts about the workshop after each session (verbally or in writing e.g., via email depending on what they feel comfortable doing).
  - The workshops will be run by trained group facilitators. We will have specific times that people with different types of experience (including those with lived experience) are given a

chance to speak. This will ensure that everyone has the chance to speak, if they want to, whilst trying to minimise the risk of dominating discussions.

- Depending on COVID-19 restrictions, workshops might take place online via Zoom. If this is the case, young people will have the opportunity to contribute via different ways e.g., verbally, via Chat, online white boards (e.g., Jamboard), depending on their preference. Young people will also be encouraged to email the research team after the session to share any thoughts they did not feel comfortable doing during the workshops.
