# Supplementary material for: Which programmes and policies across health and community settings will generate the most significant impacts for youth suicide prevention in Australia and the UK? Protocol for a systems modelling and simulation study
Source: BMJ Open. 2023 Aug 14;13(8):e071111. doi: 10.1136/bmjopen-2022-071111 (PMC10432673; doi:10.1136/bmjopen-2022-071111)
Supplement: Author's manuscript [file bmjopen-2022-071111.draft_revisions.pdf]

BMJ Open

BMJ Open is committed to open peer review. As part of this commitment we make the peer review history of every article we publish publicly available.

When an article is published we post the peer reviewers' comments and the authors' responses online. We also post the versions of the paper that were used during peer review. These are the versions that the peer review comments apply to.

The versions of the paper that follow are the versions that were submitted during the peer review process. They are not the versions of record or the final published versions. They should not be cited or distributed as the published version of this manuscript.

BMJ Open is an open access journal and the full, final, typeset and author-corrected version of record of the manuscript is available on our site with no access controls, subscription charges or pay-per-view fees (http://bmjopen.bmj.com).

If you have any questions on BMJ Open's open peer review process please email info.bmjopen@bmj.com

BMJ Open

System Dynamics Modelling for Suicide Prevention (SEYMOUR): Study Protocol

Journal:	*BMJ Open*
Manuscript ID	bmjopen-2022-071111
Article Type:	Protocol
Date Submitted by the Author:	15-Dec-2022
Complete List of Authors:	Michail, Maria; University of Birmingham<br>Robinson, Jo; Orygen The National Centre of Excellence in Youth Mental Health; The University of Melbourne Centre for Youth Mental Health<br>Witt, Katrina; Orygen The National Centre of Excellence in Youth Mental Health; The University of Melbourne Centre for Youth Mental Health<br>Occhipinti, Jo-an; The University of Sydney; Computer Simulation & Advanced Research Technologies (CSART)<br>Skinner, Adam; The University of Sydney<br>Lamblin, Michelle; Orygen The National Centre of Excellence in Youth Mental Health; The University of Melbourne Centre for Youth Mental Health<br>Veresova, Maria; Orygen The National Centre of Excellence in Youth Mental Health; The University of Melbourne Centre for Youth Mental Health<br>Kartal, Dzenana; Orygen The National Centre of Excellence in Youth Mental Health; The University of Melbourne Centre for Youth Mental Health<br>Waring, Justin ; University of Birmingham
Keywords:	Suicide & self-harm < PSYCHIATRY, MENTAL HEALTH, Health policy < HEALTH SERVICES ADMINISTRATION & MANAGEMENT

SCHOLARONE™
Manuscripts

System Dynamics Modelling for Suicide Prevention (SEYMOUR): Study Protocol

Maria Michail[1*], Jo Robinson[2,3], Katrina Witt[2,3], Jo-An Occhipinti[4,5], Adam Skinner[4], Michelle Lamblin[2,3], Maria Veresova[2,3], Dzenana Kartal[2,3], Justin Waring[6].

[1] Institute for Mental Health, School of Psychology, University of Birmingham, Birmingham, UK

[2] Orygen, The National Centre of Excellence in Youth Mental Health, Victoria, Australia.

[3] Centre for Youth Mental Health, The University of Melbourne, Parkville, Victoria, Australia.

[4] Brain and Mind Centre, Faculty of Medicine and Health, University of Sydney, Sydney, Australia

[5] Computer Simulation & Advanced Research Technologies (CSART), Sydney, Australia

[6] Health Services Management Centre, University of Birmingham, Birmingham, UK

*Corresponding author email: m.michail@bham.ac.uk

For peer review only

For peer review only - http://bmjopen.bmj.com/site/about/guidelines.xhtml

Abstract

Introduction

Suicide is a leading cause of mortality among young people aged 15-24 globally. Despite the deployment of comprehensive suicide prevention strategies, we still do not know which interventions, for which groups of young people, for how long, and with what intensity could generate the most significant reductions in suicide rates. System dynamics modelling has the potential to address these gaps. SEYMOUR will develop and evaluate a system dynamics model that will indicate which suicide prevention interventions could generate the most significant reductions in rates of suicide and attempted suicide among young people aged 12-25 in Australia and the UK.

Methods and analysis

A comparative case study design, applying participatory system dynamics modelling in North-West Melbourne (Australia) and Birmingham (UK). A computer simulation model of mental health service pathways and suicidal behaviour among young people in North-West Melbourne will be developed through three workshops with the expert stakeholder group (young people with lived experience, carers, clinicians, policy makers, commissioners). The model will be calibrated and validated using national, state, and local datasets (inputs). The simulation model will test a series of interventions identified in the workshops for inclusion. Primary model outputs include suicide deaths, self-harm hospitalisations and self-harm presentations to emergency departments. An implementation strategy for the sustainable embedding of promising suicide prevention interventions will be developed. This will be followed by model customisation, re-parameterisation, and validation in Birmingham and adaptation of the implementation strategy.

Ethics and dissemination

The project has received approval by The University of Melbourne Human Research Ethics Committee (2022-22885-25971-4), The University of Birmingham Science, Technology, Engineering and Mathematics Ethics Review Committee (ERN_21-02385) and the UK HRA (22/HRA/3826). SEYMOUR's dissemination strategy includes open-access academic publications, conference presentations, accessible findings coproduced with young people, e-briefs to policy makers, webinars for service providers and commissioners.

Strengths and Limitations

- System Dynamis Modelling (SDM) is unique in combining existing scientific evidence, empirical data, and the experiential knowledge of expert stakeholders to inform model development, evaluation, and refinement.
- SDM allows us to simulate the likely impacts of interventions over time on desired outcomes and understand with what intensity and for how long investments are required to sustain the effects of an intervention prior to implementing any changes in the real world.
- Parameter uncertainty and poor data inputs could impact model credibility and usability.

For peer review only - http://bmjopen.bmj.com/site/about/guidelines.xhtml

Introduction

Suicide is one of the leading causes of mortality among young people aged 15-24 globally (1). Self-harm and suicidal thoughts and behaviours (STB) are complex phenomena linked to a gradual and increasing build-up of vulnerability associated with multiple, interacting, and interrelated biological, psychosocial, cultural, and political factors (2). The suicide mortality rate is one of the indicators within the UN's Sustainable Development Goal (SDG) 3, and the WHO Comprehensive Mental Health Action Plan (2013-2030) identifies young people as a high-risk group setting out several key strategies for achieving the global target of reducing the rate of suicide by one-third, by 2030. These strategies include improving surveillance data, reducing access to suicide methods, reducing suicide-related stigma, access to medical services and specialist treatment, crisis intervention, and postvention.

Despite the deployment of comprehensive and multilevel suicide prevention strategies (3), we still do not know which interventions, for which groups of young people, for how long, and with what intensity could generate the most significant reductions in youth suicide rates (4). Therefore, we cannot efficiently and effectively inform decision-making in youth suicide prevention policy and planning.

The success of global and national suicide prevention strategies has been hampered by many factors including:

- a lack of involvement of young people in the design of interventions meaning that they are not youth-focused and, therefore, not acceptable to end-users (5).
- a lack of planning for the systematic uptake and implementation of strategies in the real world (6).
- limited adequacy and accuracy of current methodological approaches (e.g., meta-analyses, regression models) for synthesizing and operationalising research evidence used to inform suicide prevention strategies (7). Current methodological approaches do not: i) respond to real-time suicide data; ii) account for the interdependence of suicide risk factors as they operate across multiple levels (e.g., individual, social, health system); and iii) consider the complexity of health systems and the influence of factors such as healthcare constraints, e.g., access and capacity (8,9).

The potential of system dynamics modelling

Systems thinking and system dynamics modelling (SDM) offer a much-needed paradigm shift in suicide prevention. Systems thinking helps recognise, describe, and understand the complex and dynamic interactions within a system, where a system is conceptualised as a complex set of inter-dependent relationships between heterogenous people, organisations, or other units of analysis (10). SDM is a computer-assisted method that helps frame, test and simulate the causal processes and interactions that underlie complex systems or behaviours to inform policy making (11). SDM allows policy makers to experiment with different "*what-if*" scenarios to understand system behaviour. Most importantly, SDM allows us to simulate the likely impacts of interventions over time on desired outcomes and understand with what intensity and for how long investments are required to sustain the effects of an intervention.

As such, SDM addresses existing gaps in suicide prevention research, policy and practice in three key ways. First, SDM has the capacity to model multiple interacting and interrelated suicide risk factors (*dynamic complexity*) to understand likely trajectories of suicidal behaviour, to model the impacts of alternative interventions and thereby determine what works, for which groups of young people (e.g.,

For peer review only - http://bmjopen.bmj.com/site/about/guidelines.xhtml

males vs. females) and why. Second, SDM is grounded in the principles of implementation science, thus, maximising opportunities for policy adoption and uptake in the real world 6,8,12). Third, SDM combines existing scientific evidence, empirical data, and the experiential knowledge of stakeholders (e.g., young people, healthcare providers, service commissioners) to co-produce a computational model that simulates what happens in the real world. It is this participatory approach to model development, evaluation, and refinement that ensures sufficient implementation planning before rollout (13,14,15).

SDM has been previously adopted to inform policy and planning in mental health and suicide prevention. In Australia, SDM has been used as a decision tool to inform both regional (16,17) and national plans for suicide prevention (18). The adoption, however, of SDM in tackling the increasing suicide rates among young people is still in its infancy (4); yet its potential to efficiently and effectively guide suicide prevention strategies tailored to the unique needs of young people is great.

Aims

SEYMOUR (System Dynamics Modelling for Suicide Prevention) will develop and evaluate a SDM to inform youth suicide prevention policy, planning and implementation in Australia and the UK. Specifically, SEYMOUR aims to:

1. Develop and validate a model to inform the most appropriate combination of population-level suicide prevention interventions that would generate the most significant reductions in rates of suicide and attempted suicide among young people aged 12-25 over a 10-year period in North-West Melbourne (2023-2033).
2. Develop an implementation strategy to facilitate the adoption of the model as a decision-making tool in youth suicide prevention policy and practice in North-West Melbourne.
3. Adapt and validate the model in the UK context and optimise how it can inform policy, system-level reform, and service redesign in relation to youth suicide.

Methods and analysis

Context

SEYMOUR will adopt a comparative case study design and take place in Australia and the UK. Both countries have experienced a steady increase in suicide rates among young people aged 12-25 over the past ten years, particularly among young females (19,20). In 2021, the UK recorded the largest increase in suicide rates among females aged 24 years or under (3.6 deaths per 100,000 females) since 1981 (2.3 deaths per 100,000 females). In Australia, suicide rates for females aged 15-19 and 20-24 increased from 6.1 to 7.1 (per 100,000 females) and from 7.4 to 9.0, respectively, between 2020 and 2021.

In Australia, the project will take place in North-West Melbourne, which includes some of Victoria's most socioeconomically disadvantaged and culturally diverse areas (21). In the UK, the project will take place in Birmingham – with 40% of its population under 25 and among the most culturally diverse and socioeconomically deprived cities in the UK.

Methodology and study design

This is a mixed-methods study delivered via three inter-related work packages guided by Forrester's SDM framework (22) which involves problem definition and model conceptualisation, formulation, and testing (Aim 1); and development of an implementation strategy (Aim 2), both of which will take

For peer review only - http://bmjopen.bmj.com/site/about/guidelines.xhtml

place in North-West Melbourne, Australia. This will be followed by model customisation, re-parameterisation, and validation in Birmingham, UK, and adaptation of the implementation strategy (Aim 3).

The study is based on a participatory modelling approach which allows for a diverse group of stakeholders to be actively involved in conceptually mapping causal pathways for the development of suicidal behaviour in young people and mental health service pathways in a region (15). Stakeholders can provide unique knowledge on how the mental health system operates in their region; identify weaknesses in the system (i.e., leverage points); suggest and evaluate ideas for strategic interventions, polices or service planning decisions that would optimise the structure of the system and how it works (i.e., mechanisms of effect). Importantly, stakeholder knowledge of the local barriers and facilitators of translation of different interventions can inform the development of implementation strategies tailored to the local context (13). The unique combination of expert knowledge and empirical data has the potential to enhance model credibility and utility (23), as well as the translation of the model into effective suicide prevention policy and practice in Australia and the UK.

Sampling strategy

This sampling strategy applies across all work packages. Purposive sampling will be used to ensure maximum variation in the perspectives, cases and expertise recruited, including:

- Young people aged 12-25 years with lived/living experience of self-harm and/or suicidal behaviour (regardless of diagnosis), living within the catchment area of North-West Melbourne/Birmingham.
- Staff from non-governmental organisations (NGOs) who provide services to young people with lived/living experience of self-harm and/or suicidal behaviour, operating within the catchment area of North-West Melbourne/Birmingham.
- Family members/carers of young people aged 12-25 with lived experience of self-harm and/or suicidal behaviour, living within the catchment area of North-West Melbourne/Birmingham.
- Primary and community healthcare practitioners including General Practitioners, practice nurses, community nurses, nurse practitioners, allied health and mental health professionals working within the catchment area of North-West Melbourne/Birmingham.
- Regional suicide prevention policy leads, service planners, health and social care policy makers, operating within state of Victoria/West Midlands.

Recruitment procedures

Two expert stakeholder groups (Australia and UK) will be recruited consisting of ten to fifteen participants from the aforementioned groups.

Australia

Young people aged 12-25 will be recruited from the five headspace centres[1] operated by Orygen[2] and serving the catchment area of North-West Melbourne: Werribee, Glenroy, Craigieburn, Sunshine, and Melton. The research study team will liaise with clinical staff to determine participant

[1] headspace centres in Australia provide multidisciplinary frontline care to young people aged 12-25.
[2] Orygen is an internationally acknowledged youth mental organisation with expertise in research, policy, education, and innovations in care for young people.

For peer review only - http://bmjopen.bmj.com/site/about/guidelines.xhtml

eligibility. For young people under the age of 18, consent will be sought from parents/legal guardians.

Family members or carers of young people aged 12-25 with lived/living experience of self-harm and/or suicidal behaviour will be recruited from the same five headspace centres and local parent support groups.

Primary and community healthcare practitioners will be recruited via North-West Melbourne Primary Health Network (PHN)[3] health services. The study poster will be uploaded on the North-West PHN website and practitioners interested in being involved will contact the research team directly. Participants will also be recruited via the five headspace centres and other professional networks.

Regional suicide prevention policy leads, service planners and healthcare policy makers will be recruited across a range of professional networks and governmental organisations such as the Department of Health and Human Services, Victoria.

UK

Young people aged 12-25 as well as family members/carers will be recruited using social media and from community settings, youth clubs and parent support groups across Birmingham. Primary, secondary and community healthcare practitioners will be recruited via three National Health Service (NHS) sites: Birmingham and Solihull Clinical Commissioning Group, the largest clinically-led commissioning organisation in England, which includes 170 GP practices; Birmingham Community Healthcare NHS Foundation Trust, where we will recruit a range of health professionals including community paediatricians, school nurses, and nursing staff; Birmingham Women's and Children's NHS Foundation Trust, which hosts Forward Thinking Birmingham, the city's youth mental health service for 0-25-year-olds. NGOs staff will be recruited via social media. Regional suicide prevention policy leads, service planners and policy makers will be recruited via professional networks and governmental organisations such as the Birmingham Suicide Prevention Steering Group and the West Midlands Combined Authority.

The procedure

Work package 1: Development and validation of a youth suicide prevention SDM in North-West Melbourne, Australia

A computer simulation model of mental health service pathways and suicidal behaviour among young people will be developed through three participatory workshops with the expert stakeholder group. This work package will consist of the following phases:

Phase 1: Model conceptualisation, formulation, calibration, and validation

Model conceptualisation: Two workshops will be conducted with the expert stakeholder group. At workshop 1, the research team will introduce the basic principles of systems modelling to the participants to ensure they can meaningfully engage in the model development process. The expert stakeholder group will then conceptualise and map the pathways for the development of self-harm and suicidal behaviour in young people identifying risk and protective factors and mapping community and mental health service pathways and the factors that influence the flow of the population along these pathways. The expert stakeholder group will work together to prioritize the

---

[3] Primary Health Networks (PHNs) are independent organisations funded by the Australian Government to coordinate primary health care in their respective region.

For peer review only - http://bmjopen.bmj.com/site/about/guidelines.xhtml

interventions, policy initiatives or programs that they would like to see included in the model to capture their effects. By the end of the first workshop, the expert stakeholder group will have co-designed a draft conceptual map of the youth mental health system structure in North-West Melbourne.

At workshop 2, the research team will present the draft model structure and logic to the expert stakeholder group for review and refinement. An intervention mapping exercise will follow where participants will define and describe the prioritised interventions (e.g., components, mechanisms of effect, unintended consequences, scalability) and identify where each intervention is likely to have its effect in the model structure (*dynamic hypotheses*).

Workshops will be audio recorded and the research team will keep summary points (not ascribed to specific participants) using field notes, post-it notes, butcher's paper, whiteboard. Field notes will be analysed using codebook thematic analysis (24).

Formulation and model calibration: Between workshop 1 and 2, the research team will use the conceptual map to create a computational simulation model using Stella Architect (Version 3.1.1). The structure and parameterisation of the model will draw on national, state, and local datasets (inputs), e.g., prevalence of mental health disorder and help-seeking data (Australian Bureau of Statistics National Survey of Mental Health and Wellbeing, 2020-2021); demographic data (Australian Bureau of Statistics). Primary model outputs will include suicide deaths, self-harm hospitalisations (used as proxy for suicide attempts where such data is not captured; 4) and self-harm presentations to emergency departments. Estimates of the effects of the prioritised interventions and the mechanisms of action will be based on empirical, peer-reviewed research identified by the research team (e.g., Cochrane systematic reviews). Parameter values that cannot be derived from available data will be estimated via constrained optimisation. Powell's method (25) will be employed to obtain the set of (optimal) parameter values minimising the sum of the mean absolute percent error calculated for each time series separately (i.e., the mean of the absolute differences between the observed time series values and the corresponding model outputs, where each difference is expressed as a percentage of the observed value). The research team will also engage with key stakeholders (e.g., Victoria Department of Health) for expert advice and direction to relevant data sources that would facilitate model calibration and validation.

Model validation: Verification and validation of the simulation model will be carried out during Phase 1 to ensure the model is acceptable for its intended purpose and accurate. The expert stakeholder group will assess whether the model structure and forward projection are plausible given their knowledge of the system and local context (26).

Phase 2: User interaction with the model, policy testing and sensitivity analysis

The third workshop will be an opportunity for the research team to demonstrate the final version of the systems model structure and logic. The simulation model will incorporate a series of interventions identified in the workshops for inclusion e.g., specific suicide prevention interventions, public mental health interventions and mental health service interventions. The expert stakeholder group will interact with the tool to run a set of interventions or policy initiatives to understand the effects generated by the model as well as trade-offs between different scenarios. The research team will interpret the results of the simulated scenarios and draw out initial model insights with regards to what interventions (or combination of interventions) would generate the most significant reductions in rates of suicide, self-harm hospitalisations and self-harm presentations to emergency departments among young people aged 12-25 over a 10-year period (2023-2033) in North-West

For peer review only - http://bmjopen.bmj.com/site/about/guidelines.xhtml

Melbourne. Intervention scenarios will be compared against a baseline (business as usual), in which existing programs or policies remain in place until the end of the simulation. Sensitivity analyses will be performed to assess the impact of uncertainty in estimates of the direct effects of each intervention on the simulation results. Latin hypercube sampling will be used to draw 100 sets of values for selected model parameters determining the direct effects of the interventions on cases of hospital-treated attempted suicide and cases of suicide from a uniform joint distribution spanning +/− 20% of the default values. Differences in projected outcomes between the baseline and intervention scenarios will be calculated for each set of parameter values and summarised using simple descriptive statistics (4).

Work package 2: Development of implementation strategy of the SDM

This work package will provide evidence on the optimal strategy for the implementation and sustainable embedding of the selected suicide prevention interventions for young people in North-West Melbourne based on the modelling.

Phase 1: Contextual assessment of implementation barriers and facilitators

Phase 1 will identify the contextual and procedural factors that influence the translation and adoption of the interventions in North-West Melbourne. We will conduct qualitative interviews and focus groups with different stakeholder groups to explore a wide range of views about the benefits, barriers to, and consequences of implementing the SDM in North-West Melbourne. The topic guide (Supplementary Material 1) was informed by an existing participatory system modelling evaluation framework (27) and the Consolidated Framework for Intervention Research (CFIR; 28,29) interview guide tool to ensure a systematic and comprehensive assessment of potential barriers and facilitators of implementation of the SDM (e.g., barriers relevant to the intervention itself, barriers relevant to the inner setting, outer setting).

Participants

Participants from work package 1 will be invited to take part in work package 2. Work package 2 will also be open to new participants. We will follow the same sampling and recruitment strategy as per Methods.

Data collection and analysis

We will conduct semi-structured qualitative interviews with i) Twenty young people with lived/living experience of self-harm and/or suicidal behaviour; ii) Twelve suicide prevention policy leads and service commissioners; and iii) Twelve health and social care ministerial advisors and policy makers across Victoria.

We will conduct three focus groups (6-8 participants per focus group) with healthcare practitioners and allied health professionals across primary and community-based services across North-West Melbourne; and three focus groups with staff from local/regional NGOs.

Focus groups and interviews will be audio-recorded, transcribed, and edited to remove names to preserve participant anonymity. MAXQDA 2022 (30) will be used for data coding and analysis. Data will be analysed using framework analysis (31), a structured and rigorous process of theme-based analysis through the development of charts. Adopting framework analysis will allow the research team to work independently and then collaboratively to develop, review and refine themes and subthemes through an iterative process. This will allow the team to identify a wide range of views about the benefits, barriers to, and consequences of implementing the SDM within and across the

For peer review only - http://bmjopen.bmj.com/site/about/guidelines.xhtml

For peer review only

different stakeholder groups. The CFIR (28,29) will be adopted as a theoretical framework to help us conceptualise the findings and guide the context-specific assessment of the barriers and facilitators.

Phase 2: A context-specific implementation plan for the SDM

Phase 2 will be informed by the contextual assessment of barriers and facilitators of Phase 1 and will develop an implementation strategy for the sustainable embedding of the selected interventions/policies in North-West Melbourne based on the modelling. We will carry out two workshops with participants from Phase 1 to identify and tailor implementation strategies to mitigate barriers and leverage facilitators. The Expert Recommendations for Implementing Change (ERIC; 32) will be used to draw up a list of potential implementation strategies; and the CFIR–ERIC Matching Tool (33) will be used to facilitate the accurate choice of implementation strategies that could help address the Phase 1 CFIR-based barriers.

Participants

Ten to fifteen stakeholders drawn from Phase 1 will be recruited using purposive sampling to ensure maximum variation e.g., young people with lived/living experience of self-harm and/or suicidal behaviour; family/carers; health and social care policy makers; NGOs and primary healthcare providers.

Data collection and analysis

At workshop 1, the research team will present the findings of Phase 1 in relation to the barriers and facilitators for the implementation success of the suicide prevention interventions in our model. Participants will complete an online questionnaire, using Qualtrics, which will ask them to decide on the most important implementation barriers and associated strategies to address each chosen barrier (Supplementary Material 2). Data will be analysed using IBM SPSS Statistics (34). Counts and percentages will be used to report data on endorsement of implementation strategies. Those strategies with the highest cumulative endorsement will be selected to be included in the implementation strategy. Descriptive statistics will be used to present data on the most commonly reported variables influencing participants' rankings of the implementation strategies. Comments provided by participants in the free-text boxes will be coded by themes, which will be inductively derived based on descriptive coding of content (35).

At workshop 2, the research team will present the findings of the survey to all participants and through discussion the group will work together to agree on: i) a strategy that would guide the implementation of the suicide prevention interventions in practice across North Western Melbourne; and ii) a *"change map,"* i.e., a graphical depiction of the pathway to long-term implementation of the SDM that can be adapted and transferred to other geographical, cultural and service contexts. By the end of work package 2, we will have developed an implementation strategy to facilitate the sustainable embedding of promising suicide prevention interventions for young people in North-West Melbourne.

Work package 3: UK model customisation, re-parameterisation, validation and adaptation of implementation strategy

In this work package, we will translate and validate the computational model and implementation strategy developed in Australia into the UK context. This work package will be delivered in two phases:

For peer review only - http://bmjopen.bmj.com/site/about/guidelines.xhtml

For peer review only

Phase 1: Adaptation and validation of the Australian conceptual model

We will carry out two workshops with the UK expert stakeholder group (see Methods). At workshop 1, the research team will present the model originally developed in Australia and through discussion and debate, the conceptual model will be revised to reflect pathways and service provision relevant to the Birmingham context. An intervention mapping exercise similar to the one in work package 1 will be carried out to prioritise interventions for inclusion in the model and identify where each intervention is likely to have its effect in the model structure.

The research team will subsequently create a mathematical representation of the adapted conceptual model. The structure and parameterisation of the model will draw on secondary data sources (inputs) e.g., demographic data (Office of National Statistics); data on mental health hospitalisations, suicide attempts/deaths in Birmingham (Clinical Record Interactive Search system; Hospital Episode Statistics); and data on prevalence of mental disorder, and service capacity (Children and Young People's Health Services Data Set). The computer simulation model will run a series of population-level interventions prioritised in the workshops for inclusion to identify which combination of these interventions generates the most significant reductions in rates of suicide and attempted suicide among young people (stratified by males/females) during a 10yr period (2025-2035). Estimates of the effects of these interventions, and the mechanisms of action, will be based on empirical, peer-reviewed research identified by the team (e.g., Cochrane reviews) and informed by stakeholder feedback.

At workshop 2, the research team will demonstrate to the group the adapted model structure and logic. User interaction with the model and testing of different intervention scenarios will take place as per work package 1.

Phase 2: Adaptation of the implementation strategy for the Birmingham context

This work package will provide evidence on the optimal strategy for implementation of the SDM to inform policy making and service redesign in relation to youth suicide prevention in Birmingham. We will hold two workshops and an online survey with the expert stakeholder group.

Workshop 1 will involve presenting the findings from Phase 1 to the expert stakeholder group and sharing the implementation blueprint developed in Australia. We will explore the group's views on the barriers and facilitators for the implementation success of the adapted SDM using the CFIR interview guide tool (28). Participants will then be asked to complete an adapted version of the online survey questionnaire used in work package 2 (Phase 2) where they will be presented with a range of implementation strategies (using the CFIR-ERIC Matching Tool) and asked to rank those strategies they perceive to be more relevant and responsive to the local context.

At workshop 2, the research team will present the survey findings to the expert stakeholder group and through discussion the group will adapt and revise the implementation strategy that would guide the sustainable embedding of the promising suicide prevention interventions for young people in Birmingham.

Patient and public involvement

The conception and development of the study have been informed by input received by young people during consultations with the Institute of Mental Health Youth Advisory Group, University of Birmingham, UK. Young people advised on the participatory study design (workshops); ethical considerations and safety issues. Young people from Orygen's Youth Council have informed the dissemination and public engagement strategy.

For peer review only - http://bmjopen.bmj.com/site/about/guidelines.xhtml

Ethics and dissemination

The project has received ethics approval by The University of Melbourne Human Research Ethics Committee (2022-22885-25971-4), The University of Birmingham Science, Technology, Engineering and Mathematics Ethics Review Committee (ERN_21-02385) and the UK Health Research Authority (22/HRA/3826). The process of obtaining informed consent will be in accordance with all applicable regulatory requirements in each country including those required for consenting participants under the age of 16 in the UK and 18 in Australia. The main psychological risk is that young people may become distressed during the workshops or may disclose current suicide risk. Clear and detailed processes will be in place to manage these risks within the study's Risk Management Strategy informed by young people from the Institute of Mental Health, University of Birmingham youth advisory group and Orygen's Youth Council.

SEYMOUR's dissemination strategy includes academic publications in open access scientific journals; presentations at international conferences and scientific meetings dedicated to suicide prevention and data science; social media platforms; accessible findings (infographics, podcasts) coproduced with young people; e-briefs to policy makers and ministers, round table discussions for service providers and commissioners.

Discussion

SEYMOUR offers a novel paradigm for guiding the efficient and effective deployment of national and global suicide prevention strategies. The application of SDM to suicide prevention policy, planning and implementation is still in its infancy (36). SEYMOUR has the potential to help us unpack the "*black box*" in suicide prevention; that is, understand the dynamic complexity of the aetiological factors underlying suicidal behaviour in young people within a complex health and social care services system. SEYMOUR will bridge the gap between methodological approaches, such as participatory action research and simulation modelling, to help us address some of the most pressing and, so far, unsolved questions in suicide prevention: *What interventions can effectively and efficiently reduce the increasing rates of suicide and attempted suicide among young people?* In doing so, this programme of work has the potential to facilitate the transferability, internationalisation, and adoption of SDM as a decision-making tool in youth suicide prevention policy and practice globally to meet the mental health needs of young people and help reduce suicide and its associated burden.

For peer review only

References

1. World Health Organization. (2021). Suicide worldwide in 2019: global health estimates. Geneva. Licence: CC BY-NC-SA 3.0 IGO
2. Suicide by children and young people. National Confidential Inquiry into Suicide and Homicide by People with Mental Illness (NCISH). Manchester: University of Manchester, 2017.
3. World Health Organization. (2018). National suicide prevention strategies: progress, examples and indicators. World Health Organization. https://apps.who.int/iris/handle/10665/279765. License: CC BY-NC-SA 3.0 IGO
4. Occhipinti JA, Skinner A, Iorfino F, et al. Reducing youth suicide: systems modelling and simulation to guide targeted investments across the determinants. BMC Med 2021; 19(1):61 doi: 10.1186/s12916-021-01935-4
5. Robinson J, Bailey E, Witt K, et al. What Works in Youth Suicide Prevention? A Systematic Review and Meta-Analysis. EclinicalMedicine 2018;4-5:52-91.
6. Reifels L., Krishnamoorthy S., Kõlves K. et al. Implementation Science in Suicide Prevention. Crisis 2022: 43:1, 1-7
7. Page A, Atkinson JA, Heffernan M. Et al. Static metrics of impact for a dynamic problem: The need for smarter tools to guide suicide prevention planning and investment. Aust N Z J Psychiatry 2018; 52(7):660-667
8. Atkinson J., Page A., Wells R., et al. A modelling tool for policy analysis to support the design of efficient and effective policy responses for complex public health problems. Implement Sci 2015; 10, 26
9. Atkinson, J-A, Page, A, Prodan, A, et al. Systems modelling tools to support policy and planning. Lancet 2018; 391: 1158–1159
10. Meadows, D.H. Thinking in Systems: A Primer. Chelsea Green, White River Junction, 2008
11. Homer, J.B. and Hirsch, G.B. System Dynamics Modeling for Public Health: Background and Opportunities. Am J Public Health 2006; 96, 452-458
12. Northridge ME, Metcalf SS. Enhancing implementation science by applying best principles of systems science. Health Res Policy Syst 2016; 4, 14(1):74
13. Zimmerman L, Lounsbury DW, Rosen CS, et al. Participatory system dynamics modelling: Increasing stakeholder engagement and precision to improve implementation planning in systems. Adm Policy Ment Health 2016; 43(6):834-849
14. Freebairn L, Atkinson J, Kelly P. Simulation modelling as a tool for knowledge mobilisation in health policy settings: a case study protocol. Health Res Policy Syst 2016;21, 14(1):71
15. Freebairn L, Occhipinti J, Huntley S. Participatory methods for systems modelling of youth mental health: An implementation protocol. JIMR Res Protoc 2022; 7, 11 (2): e32988
16. Page A, Atkinson J-A, Campos W et al. A decision support tool to inform local suicide prevention activity in Greater Western Sydney (Australia). Am J Public Health 2018; 52(10):983-993
17. Skinner, A., Occhipinti, J., Song, Y., et al. Regional suicide prevention planning: A dynamic simulation modelling analysis. BJPsych Open 2021; 7(5), E157.
18. Page A, Atkinson JA, Heffernan M, et al. A decision-support tool to inform Australian strategies for preventing suicide and suicidal behaviour. Public Health Res Pract. 2017; 27(2):2721717
19. Office for National Statistics (ONS), released 06 September 2022, ONS website, statistical bulletin, Suicides in England and Wales: 2021 registrations

For peer review only - http://bmjopen.bmj.com/site/about/guidelines.xhtml

20. Australian Bureau of Statistics (2021) Causes of Death, Australia https://www.abs.gov.au/statistics/health/causes-death/causes-death-australia/latest-release#key-statistics accessed 09 November 2022
21. Department of Health, North Western Melbourne Primary Health Network (PHN), Needs Assessment Report November 2020 https://nwmphn.org.au/wp-content/uploads/2020/11/1.1-NWMPHN-Updated_Needs_Assessment_2019.pdf accessed 19 November 2022
22. Forrester J.W, Senge P.M. Tests for building confidence in system dynamics models. A.A. Legasto Jr., J.W. Forrester, J.M. Lyneis (Eds.), System Dynamics, TIMS Studies in the Management Sciences, North-Holland, New York (1980), pp. 209-228
23. Atkinson J-A, Wells R., Page A, et al. Applications of system dynamics modelling to support health policy. Public Health Res Pract 2016; 25(3):e2531531. doi: 10.17061/phrp2531531
24. Braun, V., & Clarke, V. Thematic Analysis: A Practical Guide. London: Sage 2021
25. Powell MJD. The BOBYQA Algorithm for Bound Constrained Optimization Without Derivatives. Technical repot no. DAMTP 2009/NA06. Department of Applied Mathematics and Theoretical Physics: Cambridge University, Cambridge (2009)
26. Sargent RG. Verification and validation of simulation models. J Simul. 2012; 7:12–2
27. Lee GY, Hickie IB, Occhipinti JA, et al. Participatory systems modelling for youth mental health: An evaluation study applying a comprehensive multi-scale framework. Int J Environ Res Public Health 2022;19(7):4015. doi: 10.3390/ijerph19074015
28. Damschroder LJ, Aron DC, Keith RE. Fostering implementation of health services research findings into practice: a consolidated framework for advancing implementation science. Implement Sci 2009; 7, 4:50.
29. Damschroder, L.J., Reardon, C.M., Opra Widerquist, M.A. et al. Conceptualizing outcomes for use with the Consolidated Framework for Implementation Research (CFIR): the CFIR Outcomes Addendum. Implementation Sci 17, 7 (2022). https://doi.org/10.1186/s13012-021-01181-5
30. VERBI Software. (2021). MAXQDA 2022 [computer software]. Berlin, Germany: VERBI Software. Available from maxqda.com
31. Gale NK, Heath G, Cameron E. Using the framework method for the analysis of qualitative data in multi-disciplinary health research. BMC Med Res Methodol 2013; 18, 13:117. doi: 10.1186/1471-2288-13-117
32. Powell, B.J., Waltz, T.J., Chinman, M.J. et al. A refined compilation of implementation strategies: results from the Expert Recommendations for Implementing Change (ERIC) project. Implementation Sci 10, 21 (2015). https://doi.org/10.1186/s13012-015-0209-1
33. Waltz TJ, Powell BJ, Fernández ME., et al. Choosing implementation strategies to address contextual barriers : diversity in recommendations and future directions. Implement Sci 2019;14(1):42
34. IBM Corp. (2020). IBM SPSS Statistics for Windows (Version 27.0) [Computer software]. IBM Corp.
35. Sandelowski, M. What's in a Name? Qualitative Description Revisited. Res Nurs Health 2010; 33, 77-84.
36. Occhipinti J-A., Skinner A., Doraiswamy P., et al. Mental health: build predictive models to steer policy. Nature 2021; 597, 633-636

For peer review only

Authors contributions

MM, JR, JW conceived the study idea. MM, JR, JW, JO designed the study. AS and KW provided methodological input and advice on data analysis. KW, DK, MV, ML will support data collection, analysis and implementation of the study. MM drafted the manuscript and all authors contributed to the edit of the manuscript. All authors read and approved the final version.

Funding statement

This work is supported by the European Union H2020-EU.1.3.2. MSCA-IF-GF - Global Fellowships (Grant agreement ID: 101026065) awarded to Dr Maria Michail.

JR is funded by a National Health and Medical Research Council Investigator Grant (GNT2008460) and a Dame Kate Campbell Fellowship from the Faculty of Medicine, Dentistry and Health Sciences at The University of Melbourne.

Competing interests

JO is both Head of Systems Modelling, Simulation & Data Science at the University of Sydney's Brain and Mind Centre and Managing Director of Computer Simulation & Advanced Research Technologies (CSART).

For peer review only

For peer review only - http://bmjopen.bmj.com/site/about/guidelines.xhtml

REF: 22885

Young People's Interview Topic Guide

Project: ***SEYMOUR: System Dynamics Modelling for Suicide Prevention***

Introduction

My name is XXX.
Thank you for agreeing to participate in this study.

Purpose:

"As I explained before, we are a team of researchers at Orygen, University of Melbourne and we have developed a model that can help us decide which interventions can help reduce rates of suicide & attempted suicide among young people aged 12-25 in North Western Melbourne.

To do this, we worked in partnership over the past few months with young people with lived experience of self-harm and/or suicidal behaviour, carers, health professionals and youth advocacy organisations supporting young people with lived experience of self-harm and/or suicidal behaviour. We also used data already collected through a wide variety of sources (e.g. Australia Bureau of Statistics) to help us test if and how well the model works; and, which interventions or programmes work best in reducing rates of suicide and attempted suicide among young people aged 12-25 in North Western Melbourne.

These tests showed that the most promising interventions are [enter].

What we would like to do now is ask young people for their views and perspectives of how best to put these interventions in practice; what could help or hinder the rollout of these interventions.

Themes/topics to be explored

1. Perceived value and interest in [enter intervention]
2. Specific role that young people can play in facilitating the adoption/implementation of [enter intervention]
3. Barriers to successful implementation of [enter intervention]
   Prompts: Ask about cultural/socioeconomic barriers, barriers in relation to the setting, context, organisation etc.

For peer review only - http://bmjopen.bmj.com/site/about/guidelines.xhtml

REF: 22885

4. Facilitators to successful implementation of [enter intervention]
   Prompts: Ask about facilitators in relation to the setting, context, population (i.e. young people), organisation etc.
5. Perceived successes and challenges experienced [relevant only if a young person has in the past received the intervention]
6. Recommendations for future roll-out of [enter intervention] in North Western Melbourne

Wrap-up questions

1. Is there anything else you would like to say that we have not covered?
2. Do you have any questions for me?

Thank you for taking part in this study.

Interview Topic Guide for Policy Makers, Service Planners and Ministerial Advisors

Project: SEYMOUR: System Dynamics Modelling for Suicide Prevention

---

Introduction

My name is [insert name]. Thank you for agreeing to participate.

Purpose:

We are a team of researchers at Orygen, University of Melbourne and we have developed a model that can help us decide which interventions can help reduce rates of suicide & attempted suicide among young people aged 12-25 in North Western Melbourne.

To do this, we worked in partnership over the past few months with young people with lived experience of self-harm and/or suicidal behaviour, carers, health professionals (e.g. GPs, psychiatrists, nurses), youth advocacy organisations supporting young people with lived experience of self-harm and/or suicidal behaviour; and local healthcare policy makers. We also used data already collected through a wide variety of sources (e.g. Australia Bureau of Statistics) to help us test if and how well the model works. This has

For peer review only - http://bmjopen.bmj.com/site/about/guidelines.xhtml

REF: 22885

helped us identify which interventions or programmes work best in reducing rates of suicide and attempted suicide among young people aged 12-25 in North Western Melbourne.

Based on our findings, the most promising interventions are [enter].

This project aims to the views of local/regional *suicide prevention policy leads, service commissioners; health and social care ministerial advisors and policy makers across Victoria [delete as appropriate] about the implementation and sustainable embedding of those suicide prevention interventions in North Western Melbourne. We are interested in your views on contextual, procedural and other factors that can influence the adoption and implementation of those interventions in practice.*

Do you have any questions before we start?

Themes/topics to be explored

7. Perceived value, relevance and interest in [enter intervention]
8. Specific role that [enter role of professional interviewed] can play in facilitating the adoption/implementation of [enter intervention]
9. Barriers to successful implementation of [enter intervention]
   Prompts: Ask about barriers in relation to individual, setting, context, organisation, resources, socio-political factors [where relevant], the intervention itself etc.
10. Facilitators to successful implementation of [enter intervention]
   Prompts: Ask about facilitators in relation to the individual, setting, context, organisation, resources, socio-political factors [where relevant], the intervention itself etc.
11. Perceived successes and challenges experienced [relevant only if professional interviewed has in the past been involved in the adoption/implementation of the intervention]
12. Recommendations for future roll-out of [enter intervention] in North Western Melbourne

Wrap-up questions

3. Is there anything else you would like to say that we have not covered?
4. Do you have any questions for me?

Thank you for taking part in this study.

For peer review only - http://bmjopen.bmj.com/site/about/guidelines.xhtml

REF: 22885

For peer review only

For peer review only - http://bmjopen.bmj.com/site/about/guidelines.xhtml

QUALTRICS SURVEY

SECTIONS

1. **Basic demographic information:**
    - age
    - gender
    - ethnicity
    - occupation
2. **Barriers to implementation**
   A list of barriers (identified via interviews/focus groups) will be presented along with a brief description of each barrier.
   "*Please accept or reject each barrier depending on how relevant or important you think it is*"

3. **Implementation strategies/plans**
    - For each accepted barrier, participants will be presented with a range of strategies (based on the CFIR-ERIC Matching Tool) and will be asked to select and rank those strategies/plans, from the list below, you believe to be more important in addressing the specific barrier (e.g., #1 is the top strategy).
    - A free-text box will be provided so that participants can add an explanation or any other information they wish.
4. **Feasibility, improvement opportunity, validity, difficulty, relevance**
   The final section of the survey will ask participants to report using a 3-point Likert scale (0=not influential; 1= somewhat influential; 2= extremely influential) to what extent feasibility (i.e. *can the strategy realistically be applied to the barrier*?); improvement opportunity (i.e. *will this strategy make a big impact*?); validity (i.e. *is the evidence base for the strategy compelling*?); difficulty (i.e. *what are the work and resource requirements for the strategy*?); and relevance (i.e. *does the strategy have direct relevance to the barrier*?) of each implementation strategy influenced their ranked choices.

For peer review only

BMJ Open

Which programs and policies across health and community settings will generate the most significant impacts for youth suicide prevention in Australia and the UK? Protocol for a systems modelling and simulation study.

Journal:	*BMJ Open*
Manuscript ID	bmjopen-2022-071111.R1
Article Type:	Protocol
Date Submitted by the Author:	07-Jun-2023

Complete List of Authors:	Michail, Maria; University of Birmingham<br>Robinson, Jo; Orygen The National Centre of Excellence in Youth Mental Health; The University of Melbourne Centre for Youth Mental Health<br>Witt, Katrina; Orygen The National Centre of Excellence in Youth Mental Health; The University of Melbourne Centre for Youth Mental Health<br>Occhipinti, Jo-an; The University of Sydney; Computer Simulation & Advanced Research Technologies (CSART)<br>Skinner, Adam; The University of Sydney<br>Lamblin, Michelle; Orygen The National Centre of Excellence in Youth Mental Health; The University of Melbourne Centre for Youth Mental Health<br>Veresova, Maria; Orygen The National Centre of Excellence in Youth Mental Health; The University of Melbourne Centre for Youth Mental Health<br>Kartal, Dzenana; Orygen The National Centre of Excellence in Youth Mental Health; The University of Melbourne Centre for Youth Mental Health<br>Waring, Justin ; University of Birmingham

**Primary Subject Heading**:	Public health
Secondary Subject Heading:	Health policy, Mental health, Public health
Keywords:	MENTAL HEALTH, Health policy < HEALTH SERVICES ADMINISTRATION & MANAGEMENT, Suicide & self-harm < PSYCHIATRY, Decision Making

SCHOLARONE™
Manuscripts

Which programs and policies across health and community settings will generate the most significant impacts for youth suicide prevention in Australia and the UK? Protocol for a systems modelling and simulation study.

Maria Michail[1*], Jo Robinson[2,3], Katrina Witt[2,3], Jo-An Occhipinti[4,5], Adam Skinner[4], Michelle Lamblin[2,3], Maria Veresova[2,3], Dzenana Kartal[2,3], Justin Waring[6].

[1] Institute for Mental Health, School of Psychology, University of Birmingham, Birmingham, UK

[2] Orygen, The National Centre of Excellence in Youth Mental Health, Victoria, Australia.

[3] Centre for Youth Mental Health, The University of Melbourne, Parkville, Victoria, Australia.

[4] Brain and Mind Centre, Faculty of Medicine and Health, University of Sydney, Sydney, Australia

[5] Computer Simulation & Advanced Research Technologies (CSART), Sydney, Australia

[6] Health Services Management Centre, University of Birmingham, Birmingham, UK

*Corresponding author email: m.michail@bham.ac.uk

For peer review only - http://bmjopen.bmj.com/site/about/guidelines.xhtml

Abstract

Introduction

Suicide is a leading cause of mortality among young people aged 15-24 globally. Despite the deployment of comprehensive suicide prevention strategies, we still do not know which interventions, for which groups of young people, for how long, and with what intensity could generate the most significant reductions in suicide rates. System dynamics modelling has the potential to address these gaps. SEYMOUR (*S*yst*e*m D*y*namics *Mo*delling for S*u*icide P*r*evention) will develop and evaluate a system dynamics model that will indicate which suicide prevention interventions could generate the most significant reductions in rates of suicide and attempted suicide among young people aged 12-25 in Australia and the UK.

Methods and analysis

A comparative case study design, applying participatory system dynamics modelling in North-West Melbourne (Australia) and Birmingham (UK). A computer simulation model of mental health service pathways and suicidal behaviour among young people in North-West Melbourne will be developed through three workshops with expert stakeholder groups (young people with lived experience, carers, clinicians, policy makers, commissioners). The model will be calibrated and validated using national, state, and local datasets (inputs). The simulation model will test a series of interventions identified in the workshops for inclusion. Primary model outputs include suicide deaths, self-harm hospitalisations and self-harm presentations to emergency departments. An implementation strategy for the sustainable embedding of promising suicide prevention interventions will be developed. This will be followed by model customisation, re-parameterisation, and validation in Birmingham and adaptation of the implementation strategy.

Ethics and dissemination

The project has received approval by The University of Melbourne Human Research Ethics Committee (2022-22885-25971-4), The University of Birmingham Science, Technology, Engineering and Mathematics Ethics Review Committee (ERN_21-02385) and the UK HRA (22/HRA/3826). SEYMOUR's dissemination strategy includes open-access academic publications, conference presentations, accessible findings coproduced with young people, e-briefs to policy makers, webinars for service providers and commissioners.

Strengths and Limitations

- System Dynamis Modelling (SDM) is unique in combining existing scientific evidence, empirical data, and the experiential knowledge of expert stakeholders to inform model development, evaluation, and refinement.
- SDM allows us to simulate the likely impacts of interventions over time on desired outcomes and understand with what intensity and for how long investments are required to sustain the effects of an intervention prior to implementing any changes in the real world.
- Parameter uncertainty and poor data inputs could impact model credibility and usability.

For peer review only - http://bmjopen.bmj.com/site/about/guidelines.xhtml

Introduction

Suicide is one of the leading causes of mortality among young people aged 15-24 globally (1). Self-harm and suicidal thoughts and behaviours (STB) are complex phenomena linked to a gradual and increasing build-up of vulnerability associated with multiple, interacting, and interrelated biological, psychosocial, cultural, and political factors (2). The suicide mortality rate is one of the indicators within the UN's Sustainable Development Goal (SDG) 3, and the WHO Comprehensive Mental Health Action Plan (2013-2030) identifies young people as a high-risk group setting out several key strategies for achieving the global target of reducing the rate of suicide by one-third by 2030. These strategies include improving surveillance data, reducing access to suicide methods, reducing suicide-related stigma, access to medical services and specialist treatment, crisis intervention, and postvention.

Despite the deployment of comprehensive and multilevel suicide prevention strategies (3), we still do not know which interventions, for which groups of young people, for how long, and with what intensity could generate the greatest reductions in youth suicide rates (4). Therefore, we cannot efficiently and effectively inform decision-making in youth suicide prevention policy and planning.

The success of global and national suicide prevention strategies has been hampered by many factors including:

- a lack of involvement of young people in the design of interventions meaning that they are not youth-focused and, therefore, not acceptable to end-users (5).
- a lack of planning for the systematic uptake and implementation of strategies in the real world (6).
- limited adequacy and accuracy of current methodological approaches (e.g., meta-analyses, regression models) for synthesizing and operationalising research evidence used to inform suicide prevention strategies (7). Current methodological approaches do not: i) respond to real-time suicide data; ii) account for the interdependence of suicide risk factors as they operate across multiple levels (e.g., individual, social, health system); and iii) consider the complexity of health systems and the influence of factors such as healthcare constraints, e.g., access and capacity (8,9).

The potential of system dynamics modelling

Systems thinking and system dynamics modelling (SDM) offer a much-needed paradigm shift in suicide prevention. Systems thinking helps recognise, describe, and understand the complex and dynamic interactions within a system, where a system is conceptualised as a complex set of inter-dependent relationships between heterogenous people, organisations, or other units of analysis (10). SDM is a computer-assisted method that helps frame, test and simulate the causal processes and interactions that underlie complex systems or behaviours to inform policy making (11). SDM allows policy makers to experiment with different "*what-if*" scenarios to understand system behaviour. Most importantly, SDM allows us to simulate the likely impacts of interventions over time on desired outcomes and understand with what intensity and for how long investments are required to sustain the effects of an intervention.

As such, SDM addresses existing gaps in suicide prevention research, policy, and practice in three key ways. First, SDM has the capacity to model multiple interacting and interrelated suicide risk factors (*dynamic complexity*) to understand likely trajectories of suicidal behaviour, to model the impacts of alternative interventions and thereby determine what works, for which groups of young people (e.g., males vs. females) and why. Second, SDM is grounded in the principles of implementation science,

For peer review only - http://bmjopen.bmj.com/site/about/guidelines.xhtml

For peer review only

thus, maximising opportunities for policy adoption and uptake in the real world 6,8,12). Third, SDM combines existing scientific evidence, empirical data, and the experiential knowledge of stakeholders (e.g., young people, healthcare providers, service commissioners) to co-produce a computational model that simulates what happens in the real world. It is this participatory approach to model development, evaluation, and refinement that ensures sufficient implementation planning before rollout (13,14,15).

SDM has been previously adopted to inform policy and planning in mental health and suicide prevention. In Australia, SDM has been used as a decision tool to inform both regional (16,17) and national plans for suicide prevention (18). The adoption, however, of SDM in tackling the increasing suicide rates among young people is still in its infancy (4); yet its potential to efficiently and effectively guide suicide prevention strategies tailored to the unique needs of young people is great.

Aims

SEYMOUR (System Dynamics Modelling for Suicide Prevention) will develop and evaluate a SDM to inform youth suicide prevention policy, planning and implementation in Australia and the UK. Specifically, SEYMOUR aims to:

1. Develop and validate a model to inform the most appropriate combination of population-level suicide prevention interventions that would generate the most significant reductions in rates of suicide and attempted suicide among young people aged 12-25 over a 10-year period in North-West Melbourne (2023-2033).
2. Develop an implementation strategy to facilitate the adoption of the model as a decision-making tool in youth suicide prevention policy and practice in North-West Melbourne.
3. Adapt and validate the model in the UK context and optimise how it can inform policy, system-level reform, and service redesign in relation to youth suicide.

Methods and analysis

Context

SEYMOUR will adopt a comparative case study design and take place in Australia and the UK (February 2022 – February 2025). Both countries have experienced a steady increase in suicide rates among young people aged 12-25 over the past ten years, particularly among young females (19,20). In 2021, the UK recorded the largest increase in suicide rates among females aged 24 years or under (3.6 deaths per 100,000 females) since 1981 (2.3 deaths per 100,000 females). In Australia, suicide rates for females aged 15-19 and 20-24 increased from 6.1 to 7.1 (per 100,000 females) and from 7.4 to 9.0, respectively, between 2020 and 2021.

In Australia, the project will take place in North-West Melbourne, which includes some of Victoria's most socioeconomically disadvantaged and culturally diverse areas (21). In the UK, the project will take place in Birmingham – with 40% of its population under 25 and among the most culturally diverse and socioeconomically deprived cities in the UK.

Methodology and study design

This is a mixed-methods study delivered via three inter-related work packages guided by Forrester's SDM framework (22) which involves problem definition and model conceptualisation, formulation, and testing (Aim 1); and development of an implementation strategy (Aim 2), both of which will take place in North-West Melbourne, Australia. This will be followed by model customisation, re-

For peer review only - http://bmjopen.bmj.com/site/about/guidelines.xhtml

parameterisation, and validation in Birmingham, UK, and adaptation of the implementation strategy (Aim 3).

The study is based on a participatory modelling approach which allows for a diverse group of stakeholders to be actively involved in conceptually mapping causal pathways for the development of suicidal behaviour in young people and mental health service pathways in a region (15). Stakeholders can provide unique knowledge on how the mental health system operates in their region; identify weaknesses in the system (i.e., leverage points); suggest and evaluate ideas for strategic interventions, polices or service planning decisions that would optimise the structure of the system and how it works (i.e., mechanisms of effect). Importantly, stakeholder knowledge of the local barriers and facilitators of translation of different interventions can inform the development of implementation strategies tailored to the local context (13). The unique combination of expert knowledge and empirical data has the potential to enhance model credibility and utility (23), as well as the translation of the model into effective suicide prevention policy and practice in Australia and the UK.

Sampling strategy

This sampling strategy applies across all work packages. Purposive sampling will be used to ensure maximum variation in the perspectives, cases and expertise recruited, including:

- Young people aged 12-25 years with lived/living experience of self-harm and/or suicidal behaviour (regardless of diagnosis), living within the catchment area of North-West Melbourne/Birmingham.
- Staff from non-governmental organisations (NGOs) who provide services to young people with lived/living experience of self-harm and/or suicidal behaviour, operating within the catchment area of North-West Melbourne/Birmingham.
- Family members/carers of young people aged 12-25 with lived experience of self-harm and/or suicidal behaviour, living within the catchment area of North-West Melbourne/Birmingham.
- Primary and community healthcare practitioners including General Practitioners, practice nurses, community nurses, nurse practitioners, allied health and mental health professionals working within the catchment area of North-West Melbourne/Birmingham.
- Regional suicide prevention policy leads, service planners, health, and social care policy makers, operating within state of Victoria/West Midlands.

Recruitment procedures

Two expert stakeholder groups (Australia and UK) will be recruited consisting of ten to fifteen participants from the aforementioned groups.

Australia

Young people aged 12-25 will be recruited from the five headspace centres[1] operated by Orygen[2] and serving the catchment area of North-West Melbourne: Werribee, Glenroy, Craigieburn, Sunshine, and Melton. The research study team will liaise with clinical staff to determine participant

[1] headspace centres in Australia provide multidisciplinary frontline care to young people aged 12-25.
[2] Orygen is an internationally acknowledged youth mental organisation with expertise in research, policy, education, and innovations in care for young people.

For peer review only - http://bmjopen.bmj.com/site/about/guidelines.xhtml

eligibility. For young people under the age of 18, consent will be sought from parents/legal guardians.

Family members or carers of young people aged 12-25 with lived/living experience of self-harm and/or suicidal behaviour will be recruited from the same five headspace centres and local parent support groups.

Primary and community healthcare practitioners will be recruited via North-West Melbourne Primary Health Network (PHN)[3] health services. The study poster will be uploaded on the North-West PHN website and practitioners interested in being involved will contact the research team directly. Participants will also be recruited via the five headspace centres and other professional networks.

Regional suicide prevention policy leads, service planners and healthcare policy makers will be recruited across a range of professional networks and governmental organisations such as the Department of Health and Human Services, Victoria.

UK

Young people aged 12-25 as well as family members/carers will be recruited using social media and from community settings, youth clubs and parent support groups across Birmingham. Primary, secondary and community healthcare practitioners will be recruited via three National Health Service (NHS) sites: Birmingham and Solihull Clinical Commissioning Group, the largest clinically-led commissioning organisation in England, which includes 170 GP practices; Birmingham Community Healthcare NHS Foundation Trust, where we will recruit a range of health professionals including community paediatricians, school nurses, and nursing staff; Birmingham Women's and Children's NHS Foundation Trust, which hosts Forward Thinking Birmingham, the city's youth mental health service for 0-25-year-olds. NGOs staff will be recruited via social media. Regional suicide prevention policy leads, service planners and policy makers will be recruited via professional networks and governmental organisations such as the Birmingham Suicide Prevention Steering Group and the West Midlands Combined Authority.

The procedure

Work package 1: Development and validation of a youth suicide prevention SDM in North-West Melbourne, Australia

A computer simulation model of mental health service pathways and suicidal behaviour among young people will be developed through three, one-day participatory workshops with the expert stakeholder group. The workshops will take place at a convenient location (e.g., community hall) and will be facilitated by members of the research team (MM and KW). The facilitators have significant expertise in workshop facilitation and have received the Applied Suicide Intervention Skills Training. Participants will be reimbursed for their time and travel.

This work package will consist of the following phases:

Phase 1: Model conceptualisation, formulation, calibration, and validation

Model conceptualisation: Two workshops will be conducted with the expert stakeholder group. At workshop 1, the research team will introduce the basic principles of systems modelling to the

[3] Primary Health Networks (PHNs) are independent organisations funded by the Australian Government to coordinate primary health care in their respective region.

For peer review only - http://bmjopen.bmj.com/site/about/guidelines.xhtml

participants to ensure they can meaningfully engage in the model development process. The expert stakeholder group will then conceptualise and map the pathways for the development of self-harm and suicidal behaviour in young people identifying risk and protective factors and mapping community and mental health service pathways and the factors that influence the flow of the population along these pathways. The expert stakeholder group will work together to prioritize the interventions, policy initiatives or programs that they would like to see included in the model to capture their effects. This exercise will take place as a facilitated discussion and voting in line with Freebairn et al (15). By the end of the first workshop, the expert stakeholder group will have co-designed a draft conceptual map of the youth mental health system structure in North-West Melbourne.

At workshop 2, the research team will present the draft model structure and logic to the expert stakeholder group for review and refinement. An intervention mapping exercise will follow where participants will define and describe the prioritised interventions (e.g., components, mechanisms of effect, unintended consequences, scalability) and identify where each intervention is likely to have its effect in the model structure (*dynamic hypotheses*). The identification and prioritisation of relevant interventions for modelling will be based on i) current evidence-base (i.e., empirical, peer-reviewed research identified by the research team e.g., Cochrane systematic reviews) which will be presented to the participants during the workshops; and ii) local site needs. This could include suicide-specific interventions, public health mental health interventions, mental health service planning interventions (e.g., programmes targeting access, capacity, resources, workforce) both already implemented in the real world and hypothetical interventions as both will be useful for *what-if* scenario testing (15).

Workshops will be audio recorded and the research team will keep summary points (not ascribed to specific participants) using field notes, post-it notes, butcher's paper, whiteboard. Field notes will be analysed using codebook thematic analysis (24).

Formulation and model calibration: Between workshop 1 and 2, the research team will use the conceptual map to create a computational simulation model using Stella Architect (Version 3.1.1). The structure and parameterisation of the model will draw on national, state, and local datasets (inputs), e.g., prevalence of mental health disorder and help-seeking data (Australian Bureau of Statistics National Survey of Mental Health and Wellbeing, 2020-2021); prevalence of distress (Victorian Population Health Survey); demographic data (Australian Bureau of Statistics); community mental health services (North-West Melbourne PHN). Primary model outputs will include suicide deaths, self-harm hospitalisations (used as proxy for suicide attempts where such data is not captured; 4) and self-harm presentations to emergency departments (Victoria Emergency Minimum Dataset). Estimates of the effects of the prioritised interventions and the mechanisms of action will be based on empirical, peer-reviewed research identified by the research team (e.g., Cochrane systematic reviews). Parameter values that cannot be derived from available data will be estimated via constrained optimisation. Powell's method (25) will be employed to obtain the set of (optimal) parameter values minimising the sum of the mean absolute percent error calculated for each time series separately (i.e., the mean of the absolute differences between the observed time series values and the corresponding model outputs, where each difference is expressed as a percentage of the observed value). The research team will also engage with key stakeholders (e.g., Victoria Department of Health) for expert advice and direction to relevant data sources that would facilitate model calibration and validation.

Model verification and validation: Verification and validation of the simulation model will be carried out during Phase 1 to ensure the model is acceptable for its intended purpose and accurate. The

For peer review only - http://bmjopen.bmj.com/site/about/guidelines.xhtml

model will be validated by (i) testing whether the model could replicate historic time series data across a range of key indicators (e.g., self-harm hospitalisations, emergency department (ED) presentations, suicide deaths). Outputs will be calculated every 0.875 days (the numerical integration time step, dt, will be set to one-eighth of a week; 26) over a period of 20 years, starting from 2013, permitting comparisons of model outputs with historic data from 2013 to 2023, and forecasts of the impacts of intervention/policy scenarios simulated from the time of implementation (2023) to 2033; (ii) ensuring face validity of the model structure and performance by consulting our expert stakeholder group about whether the model and/or its behaviour are reasonable. During the participatory model building process, stakeholders will interact directly with the model interface to provide feedback on its design and functionality, run scenarios, test alternative assumptions, discuss results (27).

Phase 2: User interaction with the model, policy testing and sensitivity analysis

The third workshop will be an opportunity for the research team to demonstrate the final version of the systems model structure and logic. The simulation model will incorporate a series of interventions identified in the workshops for inclusion e.g., specific suicide prevention interventions, public mental health interventions and mental health service interventions. The expert stakeholder group will interact with the tool to run a set of interventions or policy initiatives to understand the effects generated by the model as well as trade-offs between different scenarios. The research team will interpret the results of the simulated scenarios and draw out initial model insights with regards to what interventions (or combination of interventions) would generate the most significant reductions in rates of suicide, self-harm hospitalisations and self-harm presentations to emergency departments among young people aged 12-25 over a 10-year period (2023-2033) in North-West Melbourne. Intervention scenarios will be compared against a baseline (business as usual), in which existing programs or policies remain in place until the end of the simulation. Sensitivity analyses will be performed to assess the impact of uncertainty in estimates of the direct effects of each intervention on the simulation results. Latin hypercube sampling will be used to draw 100 sets of values for selected model parameters determining the direct effects of the interventions on cases of hospital-treated attempted suicide and cases of suicide from a uniform joint distribution spanning +/− 20% of the default values. Differences in projected outcomes between the baseline and intervention scenarios will be calculated for each set of parameter values and summarised using simple descriptive statistics (4).

Work package 2: Development of implementation strategy of the SDM

This work package will provide evidence on the optimal strategy for the implementation and sustainable embedding of the selected suicide prevention interventions for young people in North-West Melbourne based on the modelling.

Phase 1: Contextual assessment of implementation barriers and facilitators

Phase 1 will identify the contextual and procedural factors that influence the translation and adoption of the interventions in North-West Melbourne. We will conduct qualitative interviews and focus groups with different stakeholder groups to explore a wide range of views about the benefits, barriers to, and consequences of implementing the SDM in North-West Melbourne. The topic guide (Supplementary Material 1) was informed by an existing participatory system modelling evaluation framework (28) and the Consolidated Framework for Intervention Research (CFIR; 29,30) interview guide tool to ensure a systematic and comprehensive assessment of potential barriers and

For peer review only - http://bmjopen.bmj.com/site/about/guidelines.xhtml

facilitators of implementation of the SDM (e.g., barriers relevant to the intervention itself, barriers relevant to the inner setting, outer setting).

Participants

Participants from work package 1 will be invited to take part in work package 2. Work package 2 will also be open to new participants. We will follow the same sampling and recruitment strategy as per Methods.

Data collection and analysis

We will conduct semi-structured qualitative interviews with i) Twenty young people with lived/living experience of self-harm and/or suicidal behaviour; ii) Twelve suicide prevention policy leads and service commissioners; and iii) Twelve health and social care ministerial advisors and policy makers across Victoria.

We will conduct three focus groups (6-8 participants per focus group) with healthcare practitioners and allied health professionals across primary and community-based services across North-West Melbourne; and three focus groups with staff from local/regional NGOs.

Focus groups and interviews will be audio-recorded, transcribed, and edited to remove names to preserve participant anonymity. MAXQDA 2022 (31) will be used for data coding and analysis. Data will be analysed using framework analysis (32), a structured and rigorous process of theme-based analysis through the development of charts. Adopting framework analysis will allow the research team to work independently and then collaboratively to develop, review and refine themes and subthemes through an iterative process. This will allow the team to identify a wide range of views about the benefits, barriers to, and consequences of implementing the SDM within and across the different stakeholder groups. The CFIR (29, 30) will be adopted as a theoretical framework to help us conceptualise the findings and guide the context-specific assessment of the barriers and facilitators.

Phase 2: A context-specific implementation plan for the SDM

Phase 2 will be informed by the contextual assessment of barriers and facilitators of Phase 1 and will develop an implementation strategy for the sustainable embedding of the selected interventions/policies in North-West Melbourne based on the modelling. We will carry out two workshops with participants from Phase 1 to identify and tailor implementation strategies to mitigate barriers and leverage facilitators. The Expert Recommendations for Implementing Change (ERIC; 33) will be used to draw up a list of potential implementation strategies; and the CFIR–ERIC Matching Tool (34) will be used to facilitate the accurate choice of implementation strategies that could help address the Phase 1 CFIR-based barriers.

Participants

Ten to fifteen stakeholders drawn from Phase 1 will be recruited using purposive sampling to ensure maximum variation e.g., young people with lived/living experience of self-harm and/or suicidal behaviour; family/carers; health and social care policy makers; NGOs and primary healthcare providers.

Data collection and analysis

At workshop 1, the research team will present the findings of Phase 1 in relation to the barriers and facilitators for the implementation success of the suicide prevention interventions in our model. Participants will complete an online questionnaire, using Qualtrics, which will ask them to decide on

For peer review only - http://bmjopen.bmj.com/site/about/guidelines.xhtml

the most important implementation barriers and associated strategies to address each chosen barrier (Supplementary Material 2). Data will be analysed using IBM SPSS Statistics (35). Counts and percentages will be used to report data on endorsement of implementation strategies. Those strategies with the highest cumulative endorsement will be selected to be included in the implementation strategy. Descriptive statistics will be used to present data on the most commonly reported variables influencing participants' rankings of the implementation strategies. Comments provided by participants in the free-text boxes will be coded by themes, which will be inductively derived based on descriptive coding of content (36).

At workshop 2, the research team will present the findings of the survey to all participants and through discussion the group will work together to agree on: i) a strategy that would guide the implementation of the suicide prevention interventions in practice across North Western Melbourne; and ii) a "*change map,*" i.e., a graphical depiction of the pathway to long-term implementation of the SDM that can be adapted and transferred to other geographical, cultural and service contexts. By the end of work package 2, we will have developed an implementation strategy to facilitate the sustainable embedding of promising suicide prevention interventions for young people in North-West Melbourne.

Work package 3: UK model customisation, re-parameterisation, validation, and adaptation of implementation strategy

In this work package, we will translate and validate the computational model and implementation strategy developed in Australia into the UK context. This work package will be delivered in two phases:

Phase 1: Adaptation and validation of the Australian conceptual model

We will carry out two workshops with the UK expert stakeholder group (see Methods). At workshop 1, the research team will present the model originally developed in Australia and through discussion and debate, the conceptual model will be revised to reflect pathways and service provision relevant to the Birmingham context. An intervention mapping exercise similar to the one in work package 1 will be carried out to prioritise interventions for inclusion in the model and identify where each intervention is likely to have its effect in the model structure.

The research team will subsequently create a mathematical representation of the adapted conceptual model. The structure and parameterisation of the model will draw on secondary data sources (inputs) e.g., demographic data (Office of National Statistics); data on mental health hospitalisations, suicide attempts/deaths in Birmingham (Clinical Record Interactive Search system; Hospital Episode Statistics); and data on prevalence of mental disorder, and service capacity (Children and Young People's Health Services Data Set). The computer simulation model will run a series of population-level interventions prioritised in the workshops for inclusion to identify which combination of these interventions generates the most significant reductions in rates of suicide and attempted suicide among young people (stratified by males/females) during a 10yr period (2025-2035). Estimates of the effects of these interventions, and the mechanisms of action, will be based on empirical, peer-reviewed research identified by the team (e.g., Cochrane reviews) and informed by stakeholder feedback.

At workshop 2, the research team will demonstrate to the group the adapted model structure and logic. User interaction with the model and testing of different intervention scenarios will take place as per work package 1.

For peer review only - http://bmjopen.bmj.com/site/about/guidelines.xhtml

Phase 2: Adaptation of the implementation strategy for the Birmingham context

This work package will provide evidence on the optimal strategy for implementation of the SDM to inform policy making and service redesign in relation to youth suicide prevention in Birmingham. We will hold two workshops and an online survey with the expert stakeholder group.

Workshop 1 will involve presenting the findings from Phase 1 to the expert stakeholder group and sharing the implementation blueprint developed in Australia. We will explore the group's views on the barriers and facilitators for the implementation success of the adapted SDM using the CFIR interview guide tool (29). Participants will then be asked to complete an adapted version of the online survey questionnaire used in work package 2 (Phase 2) where they will be presented with a range of implementation strategies (using the CFIR-ERIC Matching Tool) and asked to rank those strategies they perceive to be more relevant and responsive to the local context.

At workshop 2, the research team will present the survey findings to the expert stakeholder group and through discussion the group will adapt and revise the implementation strategy that would guide the sustainable embedding of the promising suicide prevention interventions for young people in Birmingham.

Patient and public involvement

The conception and development of the study have been informed by input received by young people during consultations with the Institute of Mental Health Youth Advisory Group, University of Birmingham, UK. Young people advised on the participatory study design (workshops); ethical considerations and safety issues. Young people from Orygen's Youth Council have informed the dissemination and public engagement strategy.

Ethics and dissemination

The project has received ethics approval by The University of Melbourne Human Research Ethics Committee (2022-22885-25971-4), The University of Birmingham Science, Technology, Engineering and Mathematics Ethics Review Committee (ERN_21-02385) and the UK Health Research Authority (22/HRA/3826). The process of obtaining informed consent will be in accordance with all applicable regulatory requirements in each country including those required for consenting participants under the age of 16 in the UK and 18 in Australia. The main psychological risk is that young people may become distressed during the workshops or may disclose current suicide risk. SEYMOUR's Risk Management Strategy, informed by young people from youth advisory groups, is included in Supplementary Material 3.

SEYMOUR's dissemination strategy includes academic publications in open access scientific journals; presentations at international conferences; social media platforms; accessible findings (infographics, podcasts) coproduced with young people; e-briefs to policy makers and ministers, round table discussions for service providers and commissioners.

Discussion

SEYMOUR offers a novel paradigm for guiding the efficient and effective deployment of national and global suicide prevention strategies. The application of SDM to suicide prevention policy, planning and implementation is still in its infancy (37). SEYMOUR has the potential to help us unpack the "*black box*" in suicide prevention; that is, understand the dynamic complexity of the aetiological factors underlying suicidal behaviour in young people within a complex health and social care services system. SEYMOUR will bridge the gap between methodological approaches, such as

For peer review only - http://bmjopen.bmj.com/site/about/guidelines.xhtml

participatory action research and simulation modelling, to help us address some of the most pressing and, so far, unsolved questions in suicide prevention: *What interventions can effectively and efficiently reduce the increasing rates of suicide and attempted suicide among young people?* In doing so, this programme of work has the potential to facilitate the transferability, internationalisation, and adoption of SDM as a decision-making tool in youth suicide prevention policy and practice globally to meet the mental health needs of young people and help reduce suicide and its associated burden.

Contributorship statement

MM, JR, JW conceived the study idea. MM, JR, JW, JO designed the study. AS and KW provided methodological input and advice on data analysis. KW, DK, MV, ML will support data collection, analysis and implementation of the study. MM drafted the manuscript and all authors contributed to the edit of the manuscript. All authors read and approved the final version.

Competing interests

JO is both Head of Systems Modelling, Simulation & Data Science at the University of Sydney's Brain and Mind Centre and Managing Director of Computer Simulation & Advanced Research Technologies (CSART).

Funding statement

This work is supported by the European Union H2020-EU.1.3.2. MSCA-IF-GF - Global Fellowships (Grant agreement ID: 101026065) awarded to Dr Maria Michail. JW is funded by the European Union H2020-EU.1.3.2. MSCA-IF-GF - Global Fellowships (Grant agreement ID: 101026065). JR is funded by a National Health and Medical Research Council Investigator Grant (GNT2008460) and a Dame Kate Campbell Fellowship from the Faculty of Medicine, Dentistry and Health Sciences at The University of Melbourne. KW is funded by a National Health and Medical Research Council Investigator Grant (GNT1177787) and a Dame Kate Campbell Fellowship from the Faculty of Medicine, Dentistry and Health Sciences at The University of Melbourne. AS is supported by philanthropic funding from The Grace Fellowship. JO is funded by the National Health and Medical Research Council Centre of Research Excellence "*Reducing suicidal thoughts and behaviours in young people presenting for health care*" (1171910).

References

1. World Health Organization. (2021). Suicide worldwide in 2019: global health estimates. Geneva. Licence: CC BY-NC-SA 3.0 IGO
2. Suicide by children and young people. National Confidential Inquiry into Suicide and Homicide by People with Mental Illness (NCISH). Manchester: University of Manchester, 2017.
3. World Health Organization. (2018). National suicide prevention strategies: progress, examples and indicators. World Health Organization. https://apps.who.int/iris/handle/10665/279765. License: CC BY-NC-SA 3.0 IGO
4. Occhipinti JA, Skinner A, Iorfino F, et al. Reducing youth suicide: systems modelling and simulation to guide targeted investments across the determinants. BMC Med 2021; 19(1):61 doi: 10.1186/s12916-021-01935-4

For peer review only - http://bmjopen.bmj.com/site/about/guidelines.xhtml

5. Robinson J, Bailey E, Witt K, et al. What Works in Youth Suicide Prevention? A Systematic Review and Meta-Analysis. EclinicalMedicine 2018;4-5:52-91.
6. Reifels L., Krishnamoorthy S., Kõlves K. et al. Implementation Science in Suicide Prevention. Crisis 2022: 43:1, 1-7
7. Page A, Atkinson JA, Heffernan M. Et al. Static metrics of impact for a dynamic problem: The need for smarter tools to guide suicide prevention planning and investment. Aust N Z J Psychiatry 2018; 52(7):660-667
8. Atkinson J., Page A., Wells R., et al. A modelling tool for policy analysis to support the design of efficient and effective policy responses for complex public health problems. Implement Sci 2015; 10, 26
9. Atkinson, J-A, Page, A, Prodan, A, et al. Systems modelling tools to support policy and planning. Lancet 2018; 391: 1158–1159
10. Meadows, D.H. Thinking in Systems: A Primer. Chelsea Green, White River Junction, 2008
11. Homer, J.B. and Hirsch, G.B. System Dynamics Modeling for Public Health: Background and Opportunities. Am J Public Health 2006; 96, 452-458
12. Northridge ME, Metcalf SS. Enhancing implementation science by applying best principles of systems science. Health Res Policy Syst 2016; 4, 14(1):74
13. Zimmerman L, Lounsbury DW, Rosen CS, et al. Participatory system dynamics modelling: Increasing stakeholder engagement and precision to improve implementation planning in systems. Adm Policy Ment Health 2016; 43(6):834-849
14. Freebairn L, Atkinson J, Kelly P. Simulation modelling as a tool for knowledge mobilisation in health policy settings: a case study protocol. Health Res Policy Syst 2016;21, 14(1):71
15. Freebairn L, Occhipinti J, Huntley S. Participatory methods for systems modelling of youth mental health: An implementation protocol. JIMR Res Protoc 2022; 7, 11 (2): e32988
16. Page A, Atkinson J-A, Campos W et al. A decision support tool to inform local suicide prevention activity in Greater Western Sydney (Australia). Am J Public Health 2018; 52(10):983-993
17. Skinner, A., Occhipinti, J., Song, Y., et al. Regional suicide prevention planning: A dynamic simulation modelling analysis. BJPsych Open 2021; 7(5), E157.
18. Page A, Atkinson JA, Heffernan M, et al. A decision-support tool to inform Australian strategies for preventing suicide and suicidal behaviour. Public Health Res Pract. 2017; 27(2):2721717
19. Office for National Statistics (ONS), released 06 September 2022, ONS website, statistical bulletin, Suicides in England and Wales: 2021 registrations
20. Australian Bureau of Statistics (2021) Causes of Death, Australia https://www.abs.gov.au/statistics/health/causes-death/causes-death-australia/latest-release#key-statistics accessed 09 November 2022
21. Department of Health, North Western Melbourne Primary Health Network (PHN), Needs Assessment Report November 2020 https://nwmphn.org.au/wp-content/uploads/2020/11/1.1-NWMPHN-Updated_Needs_Assessment_2019.pdf accessed 19 November 2022
22. Forrester J.W, Senge P.M. Tests for building confidence in system dynamics models. A.A. Legasto Jr., J.W. Forrester, J.M. Lyneis (Eds.), System Dynamics, TIMS Studies in the Management Sciences, North-Holland, New York (1980), pp. 209-228
23. Atkinson J-A, Wells R., Page A, et al. Applications of system dynamics modelling to support health policy. Public Health Res Pract 2016; 25(3):e2531531. doi: 10.17061/phrp2531531
24. Braun, V., & Clarke, V. Thematic Analysis: A Practical Guide. London: Sage 2021

For peer review only - http://bmjopen.bmj.com/site/about/guidelines.xhtml

25. Powell MJD. The BOBYQA Algorithm for Bound Constrained Optimization Without Derivatives. Technical repot no. DAMTP 2009/NA06. Department of Applied Mathematics and Theoretical Physics: Cambridge University, Cambridge (2009)
26. Sterman, JD. Business Dynamics: Systems Thinking and Modeling for a Complex World. McGraw-Hill, 2000.
27. Sargent RG. Verification and validation of simulation models. J Simul. 2012; 7:12–2
28. Lee GY, Hickie IB, Occhipinti JA, et al. Participatory systems modelling for youth mental health: An evaluation study applying a comprehensive multi-scale framework. Int J Environ Res Public Health 2022;19(7):4015. doi: 10.3390/ijerph19074015
29. Damschroder LJ, Aron DC, Keith RE. Fostering implementation of health services research findings into practice: a consolidated framework for advancing implementation science. Implement Sci 2009; 7, 4:50.
30. Damschroder, L.J., Reardon, C.M., Opra Widerquist, M.A. et al. Conceptualizing outcomes for use with the Consolidated Framework for Implementation Research (CFIR): the CFIR Outcomes Addendum. Implementation Sci 17, 7 (2022). https://doi.org/10.1186/s13012-021-01181-5
31. VERBI Software. (2021). MAXQDA 2022 [computer software]. Berlin, Germany: VERBI Software. Available from maxqda.com
32. Gale NK, Heath G, Cameron E. Using the framework method for the analysis of qualitative data in multi-disciplinary health research. BMC Med Res Methodol 2013; 18, 13:117. doi: 10.1186/1471-2288-13-117
33. Powell, B.J., Waltz, T.J., Chinman, M.J. et al. A refined compilation of implementation strategies: results from the Expert Recommendations for Implementing Change (ERIC) project. Implementation Sci 10, 21 (2015). https://doi.org/10.1186/s13012-015-0209-1
34. Waltz TJ, Powell BJ, Fernández ME., et al. Choosing implementation strategies to address contextual barriers : diversity in recommendations and future directions. Implement Sci 2019;14(1):42
35. IBM Corp. (2020). IBM SPSS Statistics for Windows (Version 27.0) [Computer software]. IBM Corp.
36. Sandelowski, M. What's in a Name? Qualitative Description Revisited. Res Nurs Health 2010; 33, 77-84.
37. Occhipinti J-A., Skinner A., Doraiswamy P., et al. Mental health: build predictive models to steer policy. Nature 2021; 597, 633-636

For peer review only

For peer review only - http://bmjopen.bmj.com/site/about/guidelines.xhtml

REF: 22885

Young People's Interview Topic Guide

Project: ***SEYMOUR: System Dynamics Modelling for Suicide Prevention***

Introduction

My name is XXX.
Thank you for agreeing to participate in this study.

Purpose:

"As I explained before, we are a team of researchers at Orygen, University of Melbourne and we have developed a model that can help us decide which interventions can help reduce rates of suicide & attempted suicide among young people aged 12-25 in North Western Melbourne.

To do this, we worked in partnership over the past few months with young people with lived experience of self-harm and/or suicidal behaviour, carers, health professionals and youth advocacy organisations supporting young people with lived experience of self-harm and/or suicidal behaviour. We also used data already collected through a wide variety of sources (e.g. Australia Bureau of Statistics) to help us test if and how well the model works; and, which interventions or programmes work best in reducing rates of suicide and attempted suicide among young people aged 12-25 in North Western Melbourne.

These tests showed that the most promising interventions are [enter].

What we would like to do now is ask young people for their views and perspectives of how best to put these interventions in practice; what could help or hinder the rollout of these interventions.

Themes/topics to be explored

1. Perceived value and interest in [enter intervention]
2. Specific role that young people can play in facilitating the adoption/implementation of [enter intervention]
3. Barriers to successful implementation of [enter intervention]
   Prompts: Ask about cultural/socioeconomic barriers, barriers in relation to the setting, context, organisation etc.

For peer review only - http://bmjopen.bmj.com/site/about/guidelines.xhtml

REF: 22885

4. Facilitators to successful implementation of [enter intervention]
   Prompts: Ask about facilitators in relation to the setting, context, population (i.e. young people), organisation etc.
5. Perceived successes and challenges experienced [relevant only if a young person has in the past received the intervention]
6. Recommendations for future roll-out of [enter intervention] in North Western Melbourne

Wrap-up questions

1. Is there anything else you would like to say that we have not covered?
2. Do you have any questions for me?

Thank you for taking part in this study.

Interview Topic Guide for Policy Makers, Service Planners and Ministerial Advisors

Project: *SEYMOUR: System Dynamics Modelling for Suicide Prevention*

Introduction

My name is [insert name]. Thank you for agreeing to participate.

Purpose:

We are a team of researchers at Orygen, University of Melbourne and we have developed a model that can help us decide which interventions can help reduce rates of suicide & attempted suicide among young people aged 12-25 in North Western Melbourne.

To do this, we worked in partnership over the past few months with young people with lived experience of self-harm and/or suicidal behaviour, carers, health professionals (e.g. GPs, psychiatrists, nurses), youth advocacy organisations supporting young people with lived experience of self-harm and/or suicidal behaviour; and local healthcare policy makers. We also used data already collected through a wide variety of sources (e.g. Australia Bureau of Statistics) to help us test if and how well the model works. This has

For peer review only - http://bmjopen.bmj.com/site/about/guidelines.xhtml

REF: 22885

helped us identify which interventions or programmes work best in reducing rates of suicide and attempted suicide among young people aged 12-25 in North Western Melbourne.

Based on our findings, the most promising interventions are [enter].

This project aims to the views of local/regional *suicide prevention policy leads, service commissioners; health and social care ministerial advisors and policy makers across Victoria [delete as appropriate] about the implementation and sustainable embedding of those suicide prevention interventions in North Western Melbourne. We are interested in your views on contextual, procedural and other factors that can influence the adoption and implementation of those interventions in practice.*

Do you have any questions before we start?

Themes/topics to be explored

7. Perceived value, relevance and interest in [enter intervention]
8. Specific role that [enter role of professional interviewed] can play in facilitating the adoption/implementation of [enter intervention]
9. Barriers to successful implementation of [enter intervention]
   Prompts: Ask about barriers in relation to individual, setting, context, organisation, resources, socio-political factors [where relevant], the intervention itself etc.
10. Facilitators to successful implementation of [enter intervention]
    Prompts: Ask about facilitators in relation to the individual, setting, context, organisation, resources, socio-political factors [where relevant], the intervention itself etc.
11. Perceived successes and challenges experienced [relevant only if professional interviewed has in the past been involved in the adoption/implementation of the intervention]
12. Recommendations for future roll-out of [enter intervention] in North Western Melbourne

Wrap-up questions

3. Is there anything else you would like to say that we have not covered?
4. Do you have any questions for me?

Thank you for taking part in this study.

For peer review only - http://bmjopen.bmj.com/site/about/guidelines.xhtml

REF: 22885

For peer review only

For peer review only - http://bmjopen.bmj.com/site/about/guidelines.xhtml

QUALTRICS SURVEY

SECTIONS

1. **Basic demographic information:**
   - age
   - gender
   - ethnicity
   - occupation
2. **Barriers to implementation**
   A list of barriers (identified via interviews/focus groups) will be presented along with a brief description of each barrier.
   "*Please accept or reject each barrier depending on how relevant or important you think it is*"
3. **Implementation strategies/plans**
   - For each accepted barrier, participants will be presented with a range of strategies (based on the CFIR-ERIC Matching Tool) and will be asked to select and rank those strategies/plans, from the list below, you believe to be more important in addressing the specific barrier (e.g., #1 is the top strategy).
   - A free-text box will be provided so that participants can add an explanation or any other information they wish.
4. **Feasibility, improvement opportunity, validity, difficulty, relevance**
   The final section of the survey will ask participants to report using a 3-point Likert scale (0=not influential; 1= somewhat influential; 2= extremely influential) to what extent feasibility (i.e. *can the strategy realistically be applied to the barrier*?); improvement opportunity (i.e. *will this strategy make a big impact*?); validity (i.e. *is the evidence base for the strategy compelling*?); difficulty (i.e. *what are the work and resource requirements for the strategy*?); and relevance (i.e. *does the strategy have direct relevance to the barrier*?) of each implementation strategy influenced their ranked choices.

For peer review only

SEYMOUR STUDY

PARTICIPATORY MODELLING WORKSHOPS

RISK MANAGEMENT STRATEGY

- Briefing session where young people will complete a wellness plan including the details of their support person/clinician; triggers and strategies they use when feeling upset.
- Debriefing session where young people can reflect on the workshops and a debrief sheet containing information about the study, signposting information and contact information for suicide prevention helplines.
- Clinical support on stand-by throughout the workshops. Any potential escalation of risk will be dealt with on a case-by-case basis. Confidentiality and its limits will be explained in the Participant Information Sheet.
- If a participant becomes distressed during a workshop, they will be given the opportunity to have a break or withdraw from the study completely. If the participant appears to be extremely distressed or communicates current suicide risk, the interviewer will conduct a brief risk assessment. If the participant is not at risk, but remains distressed, the interviewer will encourage them to access support, offer to contact the participant's support person or parent/guardian, and/or help them engage in their pre-specified stress management technique/s (if relevant). If the participant is at immediate risk, the interviewer will contact emergency services (and/or, for participants under 18, their parent/guardian). A member of the research team will check-in either via email or text message within 48 hours.
- Disclosure of safeguarding concerns: If during the course of the research, the research team becomes aware of a child or vulnerable adult being at harm or at risk of harm, they will immediately notify their GP (and parent/legal guardian in case of minors) in order to follow relevant protocols to log concerns with their organisations.
- Disclosure of suicide risk (intent, thoughts, behaviour): All information obtained during the course of the research will be kept strictly confidential unless the research team has reasons to be concerned about the safety or wellbeing of a young person. If this is the case, the team will contact their GP or primary clinician; and inform parents/legal guardians in case of minors.
- Participants may experience negative power dynamics: The workshops will involve a range of experts including experts by experience (young people including minors); research experts (research team); experts in delivering services and/or supporting to young people with lived experience of suicidality. The research team will clarify the ground rules of the workshop at the beginning of each session highlighting the different and complementary types of expertise in the workshop; the importance of listening respectfully and carefully to one another; ensuring that everyone has the chance to speak if they want to and be mindful of dominating discussions. In order to manage power dynamics and facilitate the meaningful involvement of all participants and particularly those with lived experience, we will ensure that:
  - Young people will be briefed prior to the workshops and we will provide young people with the agenda beforehand to give them the opportunity to prepare.
  - Young people will have the opportunity to share their thoughts about the workshop after each session (verbally or in writing e.g., via email depending on what they feel comfortable doing).
  - The workshops will be run by trained group facilitators. We will have specific times that people with different types of experience (including those with lived experience) are given a

For peer review only

chance to speak. This will ensure that everyone has the chance to speak, if they want to, whilst trying to minimise the risk of dominating discussions.

- Depending on COVID-19 restrictions, workshops might take place online via Zoom. If this is the case, young people will have the opportunity to contribute via different ways e.g., verbally, via Chat, online white boards (e.g., Jamboard), depending on their preference. Young people will also be encouraged to email the research team after the session to share any thoughts they did not feel comfortable doing during the workshops.

For peer review only
